# Injecting drug use via femoral vein puncture: preliminary findings of a point-of-care ultrasound service for opioid-dependent groin injectors in treatment

**DOI:** 10.1186/1477-7517-9-6

**Published:** 2012-01-20

**Authors:** Richard Senbanjo, Tracey Tipping, Neil Hunt, John Strang

**Affiliations:** 1 Community Drug Services, KCA (UK), 171, Beaver Road, Ashford, Kent, TN23 7SG, UK; 2 Head Office, KCA (UK), Dan House, 44 East Street, Faversham, Kent, ME13 8AT, UK; 3 Centre for Research on Drugs and Health Behaviour, Department of Public Health and Policy, London School of Hygiene & Tropical Medicine, Keppel Street, London, WC1E 7HT, UK; 4 National Addiction Centre, Institute of Psychiatry, King's College London, 3-4 Windsor Walk, London, SE5 8AF, UK

**Keywords:** Injecting drug use, groin injecting, femoral vein damage, ultrasonography, harm reduction

## Abstract

**Background:**

Within the UK, injecting in the femoral vein (FV), often called 'groin injecting', is a serious cause of risk and harm. This study aimed to use ultrasound scanning as a means to engage groin injectors (GIs), examine their femoral injecting sites and assess their venous health, with the intention of developing improved responses.

**Methods:**

Between September 2006 and March 2009, GIs attending a network of community drug treatment centres in South East England were invited to attend an ultrasound 'health-check' clinic. This paper provides a narrative account of the scanning procedure and operation of the service, with descriptive statistical analysis of GIs who attended. The analysis uses a structured, specially-developed clinical data set that incorporates a categorisation for the severity of FV damage. Case studies using ultrasound images and a link to a video are provided to illustrate the range of presentations encountered and the categorisations used for severity.

**Results:**

A total of 160 groin scans (76 bilateral and 8 unilateral) were performed in 84 GIs. The majority were men (69.0%) and the mean age of the sample was 36.8 years. The mean duration of drug use and injecting drug use was 19.7 years and 13.8 years, respectively. FV damage at the injecting site in the right groin was graded as minimal in 20 patients (25%), moderate in 27 (33.8%), severe in 16 (20.0%) and very-severe in 17 (21.3%). Corresponding figures for left FV were 24 (30.0%), 22 (27.5%), 18 (22.5%) and 16 (20.0%). Wide variation was observed in the time to the development of these grades of FV damage.

**Conclusions:**

Modern, portable ultrasound scanners make it possible to examine the venous health of GIs in community treatment settings. Ultrasound scanning identified extensive FV damage, much hitherto-unrecognised in this population. These findings should further alert clinicians, policy-makers and patients to the urgent need for effective harm reduction responses to GI behaviour. Images of damaged FV in this paper might prove to be a useful resource for discussions about GI risks.

## Background

It is estimated that between 100,000 to 150,000 people currently inject drugs in England [1,2]. Investigations of samples from this population suggest that between a third and one half use the femoral vein (FV) in the groin as their main injecting site [3-5]. Groin injecting (GI) is associated with significant risks of injury to the FV and femoral artery (FA) and bacterial and blood borne viral infections [6]. More serious medical complications such as deep vein thrombosis (DVT), pulmonary embolism, chronic venous disease (CVD) and compromise to the adjacent FA with consequent risk of gangrene have also been reported [7-10]. Groin injectors (GIs) are often unaware of these risks and consequently, tend to present late for treatment of injecting complications [5]. Hospitalisation of drug users for infections and vascular damage caused by injecting drug use (IDU) has recently increased in England [11]. Cessation of GI is related to greater treatment retention, but is also a function of more severe venous disease [12]. This suggests that innovative approaches are needed, which can better engage people who inject in the FV, so that problems can be recognised and responded to more quickly and earlier cessation of GI can be promoted.

Ultrasonography is a non-invasive procedure for investigating venous and arterial diseases [13]. Ultrasound scanners use high frequency sound waves to produce two-dimensional anatomical images and spectral tracings that can demonstrate vein damage (scarring, narrowing or blockage by blood clot), arterial damage (e.g. aneurysm) as well as changes in venous and arterial blood flow. Ultrasonography is safe and the latest scanners are low cost, portable and capable of producing clear images [14,15]. Ultrasound scanners are increasingly being used by non-radiologists to enhance the speed and accuracy of clinical examinations [16] but the benefits of the technology are yet to be evaluated in drug treatment settings.

Some drug treatment services offer harm reduction advice and information including leaflets to GIs [17,18], but little is known about their effectiveness [19,20]. In isolation, simple advice and information that cautions against GI may have limited efficacy. We hypothesized that ultrasound scanning might better engage injecting drug users in discussion about GI risks and help to improve our understanding of the patho-physiology of FV damage and CVD among GIs.

The new point-of-care ultrasound service was established to provide current and former GIs with easy access to ultrasound assessment of anatomical and functional status of FV segment at their groin injecting site(s). Referral pathways to relevant medical and surgical interventions were identified for patients experiencing complications of GI. This paper describes:

1. How the service was established, the operation of the service, the scanning procedure and the characteristics of clinic attendees.

2. The clinical presentation, ultrasound findings and a grading system for the types of FV damage identified among GIs.

## Methods

### The ultrasound equipment

A Macromaxx™ (SonoSite Inc., Bothell, WA, USA) portable ultrasound scanner was purchased along with 2 broad-band transducers - a high frequency (5 - 10 MHz) flat linear array probe (L38e) and a low frequency (2 - 5 MHz) curved linear array probe (C60e). The scanner weighs 3.9 kg with the battery and the C60e transducer attached. Direct costs of the equipment in 2006 were £23,500 ($40,000).

### Operation of the service

The service was offered as a 'health check' for opioid-dependent GIs attending a network of community-based drug treatment centres in South East England. Posters and information leaflets based on the recommendations of the Royal College of Radiologists [21] were displayed in clinic waiting rooms. We developed a scanning protocol in line with the recommendations of the British Medical Ultrasonography Society [14], new forms for recording relevant clinical data and a chaperone policy. Enquiry about GI occurs at assessment and patients who wish to be scanned attend the ultrasound clinic at the drug service centre. Forty minutes is allocated for the interview, scanning and the 'feedback' of ultrasound findings; and the person's key worker generally serves as the chaperone. Verbal and written information are provided and patient participation is voluntary. Written consent is obtained prior to examination of the groin, limbs and the scanning.

### The scanning procedure and ultrasound anatomy of the femoral vessels

**Figure 1:**
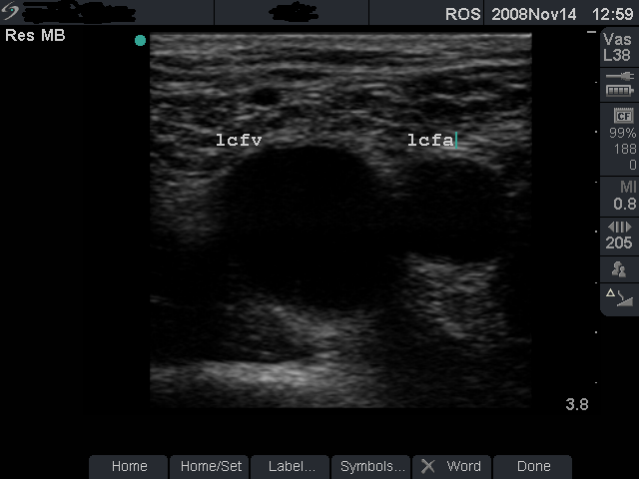
**Vein with minimal damage (grade 1)**. B-mode image of the left femoral vein (lcfv) and artery (lcfa) in a 30-year old groin injector (case study B). The damage to this vein is minimal (grade 1).

**Figure 2:**
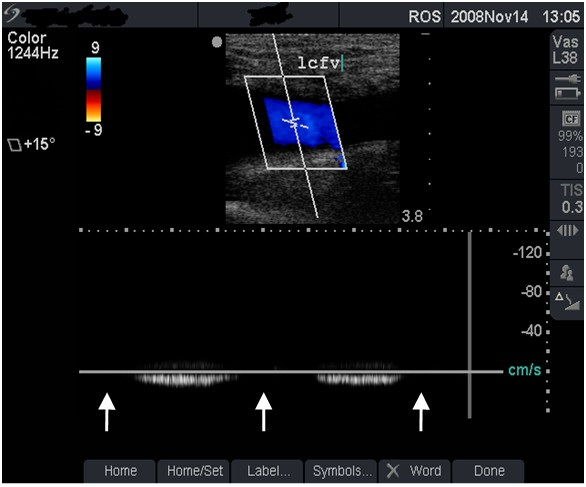
**Normal venous flow (grade 1)**. Duplex scan showing normal blood flow - colour flow (top half) and spectral tracing (lower half) in the left femoral vein (lcfv) of the patient in figure 1 (case study B). Note the normal, phasic venous flow with respiration (arrows).

**Figure 3:**
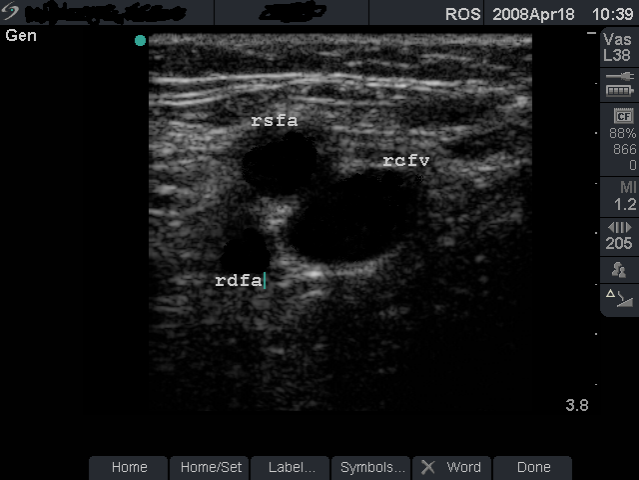
**Femoral artery anterior to femoral vein**. B-mode image taken 3 cm below the right inguinal ligament in a 24-year old groin injector. Note that the femoral artery has divided into the femoral artery of the thigh (rsfa) and the profundus (deep) artery of the thigh (rdfa).

**Figure 4:**
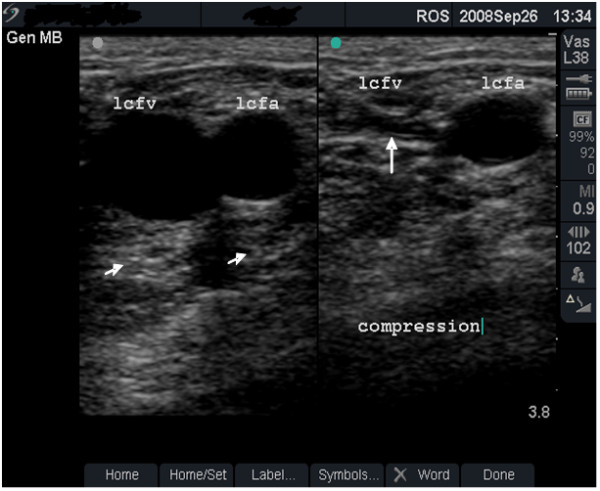
**Fully compressible, normal vein (grade 1)**. Dual-frame image of the left femoral vein (lcfv) and artery (lcfa) in a 44-year old groin injector who has never injected in the lcfv. Patency of the lcfv is demonstrated by the complete collapse of the vein with transducer pressure (long arrow). Note the post-acoustic shadows (short arrows).

**Figure 5:**
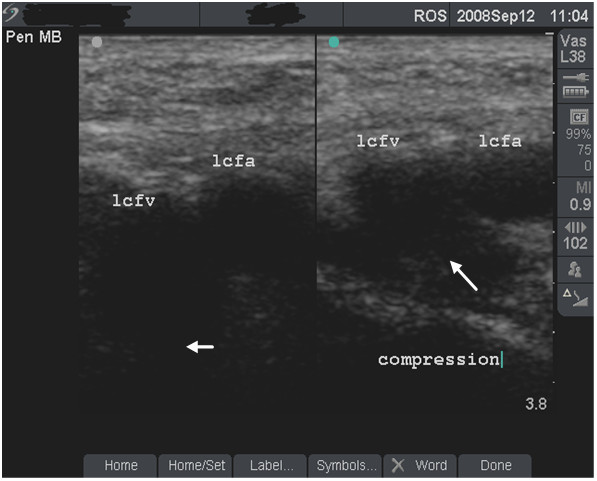
**Non-compressible vein (dual frame)**. Dual-frame image of the left femoral vein (lcfv) and artery (lcfa) in a 31-year old woman (case study A). The lcfv is non-compressible (long arrow) suggestive of acute deep vein thrombosis (DVT). Note the enhanced echo pattern in the soft tissue caused by oedema and the absence of post-acoustic shadow (small arrow).

**Figure 6:**
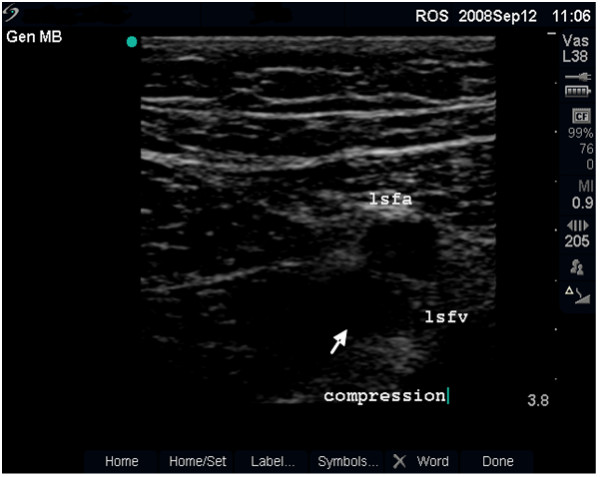
**Non-compressible vein (B mode)**. B-mode image of a non-compressible left femoral vein (lsfv, see arrow) behind the femoral artery (lsfa) in the thigh of the patient in figure 5 (case study A), indicative of extension of the blood clot to the femoral vein in the mid-thigh.

Scanning is performed with the patient lying on a couch, the body and head raised up and the limb to be examined flexed and rotated at the hip in a slight 'frog-leg' position. Coupling gel is applied and scanning starts at the GI site with the L38e probe placed lightly over the skin in the transverse plane. The size and anatomical arrangement of the femoral vessels are noted. In the groin, the FV is normally larger, medial and at the same depth as the FA (Figure 1). With the transducer turned into the longitudinal plane, blood flow is assessed at the GI site using the duplex or colour Doppler functions of the scanner (Figure 2). The probe is then allowed to slide gradually downwards (in the transverse plane) taking note of important landmarks: the sapheno-femoral junction (image not shown) and the level of the division of the FA. After dividing, the FA tends to roll on top of (anterior to) the FV (Figure 3). Starting from the groin, the probe is pressed into the skin at intervals of 2 cm to check for FV patency. Full compression should be possible with normal veins (Figure 4) and any residual non-compression may indicate presence of blood clot (Figures 5 and 6 and case study A). In the middle third of the thigh, the FV lies deep (behind) the FA (Figure 6). A video of the scanning procedure may be viewed online at http://www.kcl.ac.uk/iop/depts/addictions/research/drugs/FemoralInjecting.aspx

This video file illustrates aspects of the clinical consultation within the ultrasound 'health check' clinic. Please note that there are sequences with no sound.

### Grading of femoral vein damage (from saved ultrasound images)

FV damage in each groin is scored as one of four grades. These are based on the anatomical size and compressibility of the vein and the blood flow.

**• Grade 1 (minimal or no change) **- FV is larger than the FA (Figure 1), shows normal flow rate and pattern (Figure 2) and is fully compressible (Figure 4 and case study B).

**Figure 7:**
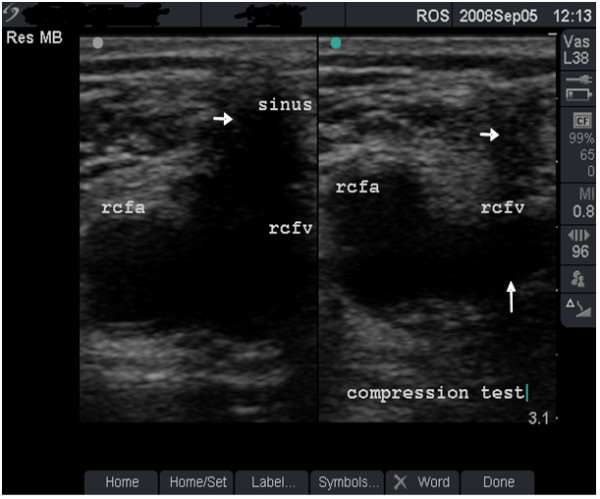
**Vein with moderate damage (grade 2)**. Dual-frame image and compression test in a 36-year old man (case study C). A sinus (short arrows) is seen above the right femoral vein (rcfv) which is not fully compressible (long arrow). This vein damage was graded as moderate (grade 2).

**Figure 8:**
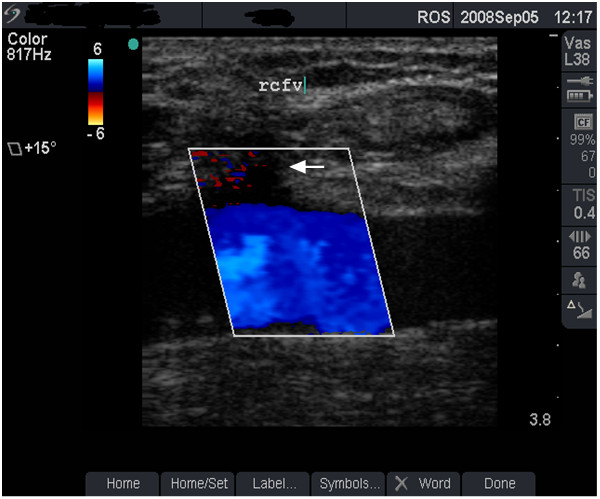
**Venous flow with minor abnormality (grade 2)**. Colour flow image from the right femoral vein (rcfv) of the patient in figure 7 (case study C). Note that venous blood flow (as indicated in colour flow image) beneath the sinus remains satisfactory (arrow).

**• Grade 2 (moderate damage) **- FV is about the same size as the FA (Figure 7), fully or partially compressible and shows minor abnormalities in flow rate or pattern (Figure 8 and case study C).

**Figure 9:**
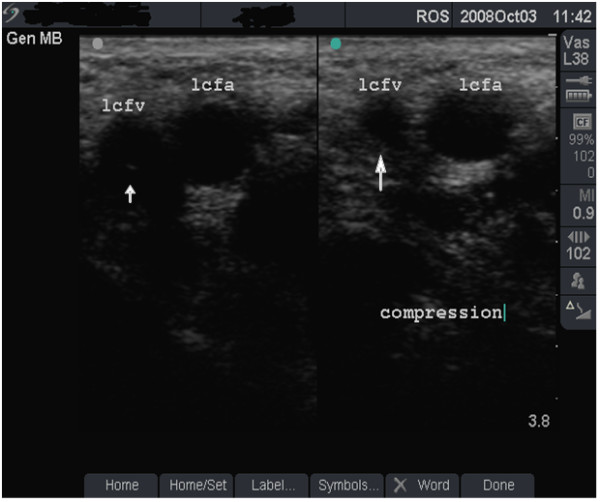
**Vein with severe damage (grade 3)**. Dual-frame image and compression test in a 43-year old woman (case study D). The left femoral vein (lcfv, short arrow) is smaller than the artery (lcfa) and the vein did not collapse completely with compression (long arrow). The damage to this vein was graded as severe (grade 3).

**Figure 10:**
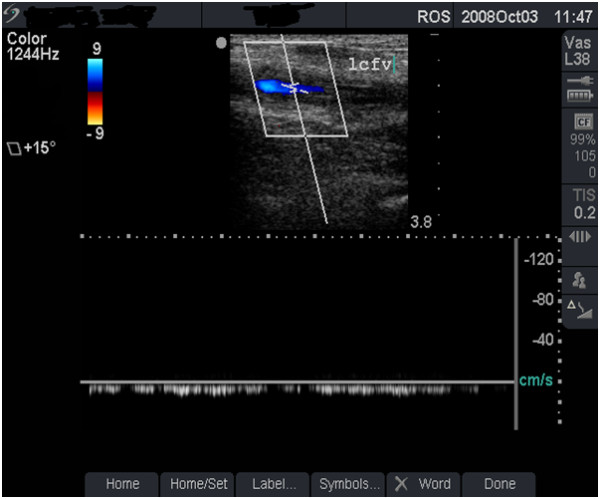
**Vein with reduced, continuous flow (grade 3)**. Duplex scan showing significant reduction in blood flow (top half of image) and continuous venous flow (lower half of image) in the severely damaged left femoral vein (lcfv) of the patient in figure 9 (case study D). Compare this reduced, continuous flow with the normal venous flow pattern in figure 2.

**• Grade 3 (severe damage) **- FV is smaller than the FA (Figure 9) and shows significant reduction in flow rate and a continuous flow pattern (Figure 10 and case study D).

**Figure 11:**
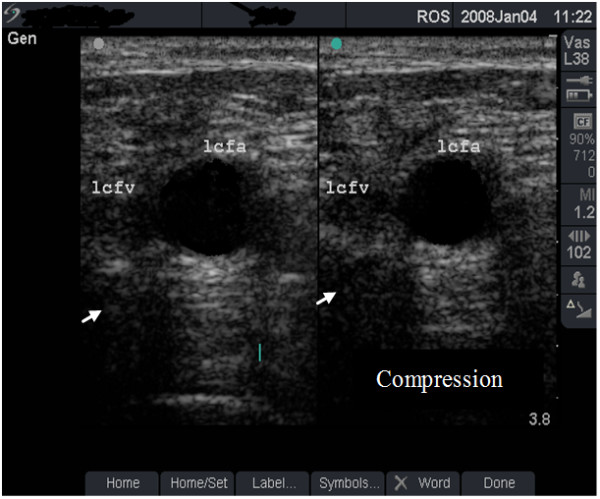
**No blood flow, very severe damage (grade 4)**. Dual-frame image from a 37-year old groin injector (see case study E). The left femoral vein (lcfv) is small and occluded by an echogenic material (organised blood clot). Note the absence of post-acoustic shadow behind the vein (arrows). The damage was graded as very-severe (grade 4).

**Figure 12:**
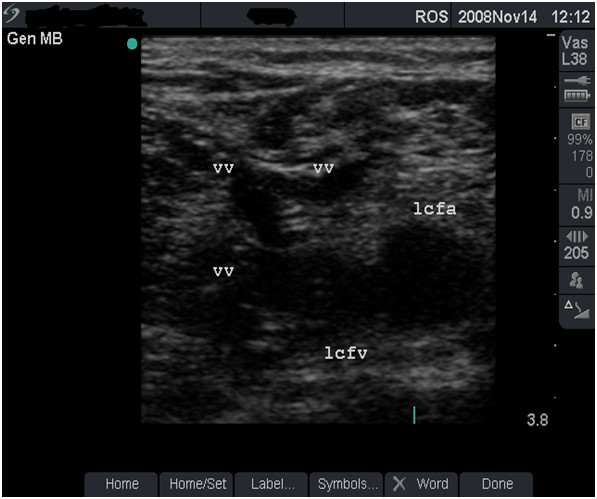
**Alternative venous drainage, very severe damage (grade 4)**. B-mode image showing a very-severely damaged left femoral vein (lcfv) and new vessels (vv), providing alternative routes for venous drainage.

**• Grade 4 (very-severe damage) **- FV is much smaller than the FA (Figure 11). Blood flow may not be detected in the sclerosed vein but may be present in collateral veins (Figure 12 and case study E).

## Results

### Initial uptake of the service

Between September 2006 and March 2009, 84 of 86 (97.7%) GIs had femoral ultrasound scan in the 'health-check' clinic. Two clinic attendees preferred not to have the scan. Scanning was bilateral in the majority (90.5%) but 8 patients (9.5%) who had injected drugs in one groin but has never done so in the other groin had unilateral scan. A total of 160 groin scans have thus been conducted.

### Patient characteristics

The participants were predominantly male (69.0%) and white European (96.4%). The mean ± SD (range) age, duration of drug use and IDU were 36.8 years ± 8.6 (21 - 56), 19.7 years ± 8.3 (4 - 39) and 13.8 years ± 7.9 (2 - 33), respectively. Opioid substitution treatment (OST) was in the form of methadone oral solution (75/84, 89.3%) or sub-lingual buprenorphine as Subutex or Suboxone (7/84, 8.3%). Two patients (2.4%) commenced OST after the scanning. Mean time in treatment was 1.8 years (SD 2.3, range 0 - 10). Of the 84 participants, 37 (44.0%) had not injected in the FV for at least one month and 47 (56.0%) reported ongoing drug use by GI. Table 1 shows the drugs 'ever' injected and the drugs currently being injected in the FV.

**Table 1:** Drugs used by injection and the injecting site among 84 clinic attendees

Substance	Route used for self administration by injection		
		**Current injection drug use**	

	**Ever injected in the groin** **N (%)**	**Injection in the groin** **N (%)**	**Injection in surface vein** **N (%)**

Heroin (n = 84)	84 (100.0%)	47 (56.0%)	8 (9.5%)

Cocaine (Crack/powder, n = 83)	59 (71.1%)	20 (24.1%)	5 (6.0%)

Snowball (combined heroin and cocaine, n = 76)	40 (52.6%)	14 (18.4%)	1 (1.3%)

Amphetamines (n = 69)	18 (26.1%)	-	-

Benzodiazepines (crushed tablets and/or ampoules, n = 69)	8 (11.6%)	-	-

Methadone ampoules (n = 69)	4 (5.8%)	-	-

Crushed tabs (n = 69)			

Codeine	3 (4.3%)	-	-

Diconal (dipipanone/cyclizine)	3 (4.3%)	-	-

Ecstasy	2 (2.9%)	-	-

Buprenorphine (Temgesic)	1 (1.4%)	-	-

Palfium	1 (1.4%)	-	-

Alcohol (n = 69)	1 (1.4%)	-	-

### Clinical presentation

The presenting symptoms and the physical findings were as shown in table 2. Half of the sample (47.6%) reported previous history of DVT in either leg. Leg pain (54.8%) and swelling (36.9%) were the most common symptoms and the majority (61.9%) had depressed scarring (sinus) in either groin.

**Table 2:** Medical history, presenting symptoms and clinical findings among 84 groin injectors

	Right (n = 80) N (%)	Left (n = 80) N (%)	Either (n = 84) N (%)
**Medical history**			

Lower limb DVT	30 (38.0%)	32 (41.0%)	39 (47.6%)

**Presenting symptoms**			

Leg pain	31 (38.8%)	28 (35.0%)	46 (54.8%)

Leg swelling	23 (28.8%)	17 (21.3%)	31 (36.9%)

**Physical signs**			

**Groin**			

Puncture/track mark	12 (15.0%)	9 (11.3%)	10 (11.9%)

Flat scar	21 (26.3%)	16 (20.0%)	17 (20.2%)

Depressed scar	43 (53.8%)	43 (53.8%)	52 (61.9%)

**Leg**			

Varicose veins	16 (20.1%)	18 (22.6%)	23 (27.4%)

Oedema	12 (15.0%)	7 (8.8%)	15 (17.9%)

Leg ulcer (open/healed)	6 (7.5%)	3 (3.8%)	7 (8.3%)

### Ultrasound scan findings

Of the 80 right groin scans, FV damage was graded as minimal in 20 (25.0%), moderate in 27 (33.8%), severe in 16 (20.0%), and very-severe in 17 (21.3%). The corresponding figures for the left groin were 24 (30.0%), 22 (27.5%), 18 (22.5%) and 16 (20.0%). No evidence of aneurysm or abnormal blood flow in the FA was detected in this sample.

### Time to development of FV damage

Time to FV damage was estimated systematically on the basis of the clinical history. Duration since first GI was noted and then adjusted to reflect subsequent periods (months) of GI cessation e.g. during imprisonment or in connection with OST or detoxification. Estimated duration varied widely, ranging from 1 to 116 months (median, 31 months) for GIs with minimal damage. The corresponding figures for moderate, severe and very-severe FV damage were 6 to 234 months (48 months), 9 to 180 months (60 months) and 12 to 240 (48 months) respectively.

### Case studies

#### Case study A

A 31-year old woman presented in the clinic with 2-week history of pain and swelling in her left lower limb for which she was prescribed antibiotics. She has been injecting heroin regularly in her left groin for one year. Ultrasound scan revealed a non-compressible left FV at the injecting site (Figure 5) suggestive of acute DVT with extension of the blood clot to the FV in the middle third of the thigh (Figure 6). She was immediately referred to the Accident and Emergency department.

#### Case study B

This asymptomatic 30-year old man has been injecting heroin intermittently in the left groin for 5 years. Ultrasound scan showed a normal-sized, fully compressible FV (Figure 1) with normal venous flow rate and pattern. The 'phasic' flow pattern in Figure 2 indicates absence of outflow obstruction from the vein. The damage to this FV was graded as minimal. Personalised feedback on femoral anatomy and the risks of GI were provided with clear advice to stop injecting drug use.

#### Case study C

An asymptomatic 36-year old man who has been injecting heroin and cocaine separately and in combination (snowball) in the right groin for 12 years. The dual image in Figure 7 demonstrates a depressed scar (sinus) at the injecting site with satisfactory blood flow through the FV (Figure 8). The damage to this FV was graded as moderate. Risk of further damage with persistent GI was explained and he was advised to stop GI.

#### Case study D

This 43-year old woman stopped injecting in the groin following her second hospital admission for left lower limb DVT, one year before she attended the clinic. Over a 15-year period, she had injected heroin, cocaine, amphetamines, and crushed diconal (dipipanone/cyclizine) and diazepam tablets in her groin. The FV is much smaller than the artery (Figure 9) with reduced blood flow and a 'continuous' flow pattern (Figure 10) indicating partial outflow obstruction. This FV was graded as severely damaged. Her feedback included an explanation of her leg ache/pain.

#### Case study E

A 37-year old man who has been injecting heroin, cocaine and 'snowball' in both groins for 4 years and is on life-long warfarin treatment for recurrent bilateral DVT. He reported having difficulties with 'finding' the FV in his groin. Figure 11 shows a small, sclerosed FV containing echogenic material (organised blood clot) with no blood flow. The vein damage was graded as 'very-severe'. The findings convinced him of the futility of further GI attempts. New venous 'channels' are often seen in patients with severely damaged FV (Figure 12).

## Discussion

Ultrasound scanning identified significant FV damage in three quarters (72.5%) of the groins examined and two-fifths (41.8%) of the veins showed 'severe' or 'very-severe' damage. Much of the FV damage and functional (blood flow) impairment identified among our sample of symptomatic and asymptomatic GIs remain under-appreciated in terms of its magnitude and impact on drug users. Vein damage at the GI site has been attributed to direct trauma from repeated vein puncture [3], irritating effects of intravenous and peri-vascular injection of substances [22,23] and septic thrombo-phlebitis and other local bacterial infections [11]. Future studies should examine the factors associated with more rapid FV damage in view of the wide variation in the time to FV damage observed among GIs in this study and by other authors [3].

Half of our sample of GIs reported past history of DVT. The observed association between GI and DVT is widely recognised in the literature [6,24]. DVT and pulmonary embolism are amongst the most serious complications of GI [9]. In addition to the risk of death from pulmonary embolism, untreated or poorly-treated DVT often results in sclerosis, narrowing or loss of patency of veins [3,6]. Sclerosed veins lose their ability to expand to accommodate increases in venous return during periods of increased physical activity. Symptoms of venous insufficiency (leg ache/pain or swelling) were reported by more than half (54.8%) of GIs in our study. These symptoms occur at a younger age among GIs compared with the general population [7,24]. The mean age of our sample of GIs was 36.8 years.

People who inject drugs in the groin often require basic information and education about femoral anatomy and the symptoms of DVT. These factors may contribute to the reported late presentation for treatment of injecting-related complications [5,11]. Our early experiences of point-of-care ultrasonography suggest that personalised feedback of ultrasound findings may enhance patient and key worker (chaperone) awareness of GI risks. Longer term evaluation of the effectiveness of this type of intervention is needed. Evaluations of the feasibility and consideration of ethical issues will be required in order to establish the parameters for any future use of ultrasound scanners to locate 'safer' surface veins in GIs who cannot be persuaded to stop injecting drug use.

Ultrasound assessment of FV damage within drug clinics has training implications. Diagnostic accuracy of ultrasonography is dependent on the experience of the operator [14,16]. Our experience suggests that point-of-care ultrasound services for injecting drug users have potential use for detecting FV damage at an earlier stage, rather than as an alternative to hospital-based radiology services. Point-of-care ultrasound service for GIs should therefore be used for the purposes of attracting, assessing and educating drug users about GI risks; and potentially, for promoting behaviour change. Patients presenting with symptoms, signs or ultrasound findings suggestive of DVT should immediately be referred to acute or emergency services.

Provision of a clinical health check with basic ultrasound scan of femoral vessels is evidently feasible within community-based drug clinics. The participants were concerned about GI risks and keen to identify the extent of any existing FV damage. By providing access to ultrasonography within familiar settings, we were able to attract and engage GIs in detailed assessment of their venous health. It is unlikely that most of these GIs would otherwise have presented to hospital radiology department for this assessment. Ultrasound scanning within drug clinics might thus provide greater opportunities for improving our understanding of GI practices and the development of effective responses to the harm associated with the behaviour.

## Conclusions

The strength of ultrasound imaging lies in its safety, non-invasive nature and comparatively low cost. Such vascular ultrasonography delivered at the point-of-care for injecting drug users has identified extensive damage, much hitherto-unrecognised, which has alerted both patients and clinicians to issues requiring urgent attention. The new clinical service has proved popular and may be a valuable tool for detecting morbidity at an early stage. Longer term evaluation of its effectiveness as a harm reduction intervention among patients who inject in the FV is now needed.

## Competing interests

All authors have no declarations of interest regarding the subject of the paper. In the wider addictions field, JS has received research funding from, and provided consultancy to, various pharmaceutical companies about new medications potentially applicable in the treatment of addictions and related complications.

## Authors' contributions

RS led the clinical work and drafting of the manuscript. TT provided managerial support for the establishment and operation of the clinic. NH and JS contributed to the drafting of the manuscript. All authors read and approved the final manuscript.
